# Self-Portraits: Smartphones Reveal a Side Bias in Non-Artists

**DOI:** 10.1371/journal.pone.0055141

**Published:** 2013-02-06

**Authors:** Nicola Bruno, Marco Bertamini

**Affiliations:** 1 Dipartimento di Neuroscienze, Università di Parma, Parma, Italy; 2 Department of Experimental Psychology, University of Liverpool, Liverpool, United Kingdom; CSIC-Univ Miguel Hernandez, Spain

## Abstract

According to surveys of art books and exhibitions, artists prefer poses showing the left side of the face when composing a portrait and the right side when composing a self-portrait. However, it is presently not known whether similar biases can be observed in individuals that lack formal artistic training. We collected self-portraits by naïve photographers who used the iPhone™ front camera, and confirmed a right side bias in this non-artist sample and even when biomechanical constraints would have favored the opposite. This result undermines explanations based on posing conventions due to artistic training or biomechanical factors, and is consistent with the hypothesis that side biases in portraiture and self-portraiture are caused by biologically- determined asymmetries in facial expressiveness.

## Introduction

When they compose a self-portrait, artists prefer poses showing the right side of their face [1]–[5]. This right-side bias is well documented by surveys of art books and exhibitions but its origin has remained controversial [6]. As an alternative to observational data from the history of art, we collected self-portraits by naïve photographers who used the iPhone™ front camera in controlled settings. The right side bias remained observable in this non-artist sample, and even when biomechanical constraints would have favored a left-side bias. These results argue against explanations based on posing constraints and support the hypothesis that side biases in portraiture and self-portraiture are caused by biologically determined asymmetries in facial expressiveness [7].

**Figure 1:**
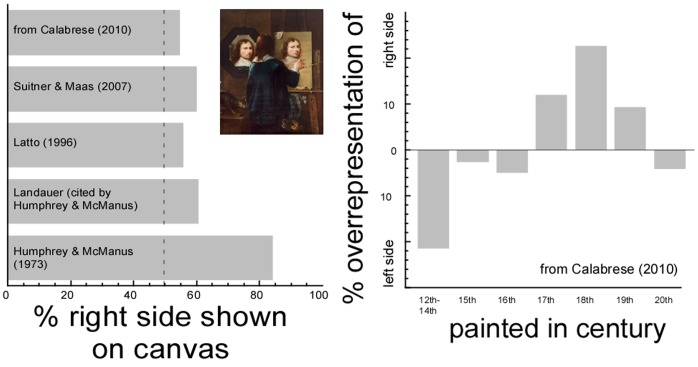
Artists prefer poses showing the right side of their face when composing a self-portrait. (a) A synopsis of available data on side biases in self-portraits by artists, including, three published studies (1–3), an unpublished study (4), and our own unpublished analysis based on illustrations in a recent monograph (5). Right-handed artists may have found it easier to copy from a mirror placed on the left of the canvas, as in a famous self-portrait by Dutch painter Johannes Gummp (inset). Alternatively, artist may have sought studio arrangements that allowed them to display their left, more expressive side. (b) Percent over-representations of the right (left) side were computed by taking the ratio frequency (right-sided)/total three quarter poses and then subtracting this from 0.5 (expected if there is no bias), within each temporal bin. Thus, negative values signify an over-representation of left-sided portraits, positive values of right-sided ones.

Figure 1a presents a synopsis of the available data on side biases in self-portraiture by artists. We were able to identify five sources of such data. Three were in published papers [1]–[3]. One consisted of unpublished results cited in one of these papers [4]. A fifth source of data came from a recently published monograph on the semiology of self-portraits [5]. Because this last source included a rich selection of works from the Middle Ages up to the twentieth century, we decided to analyze this body of self-portraits for inclusion in our literature review. All the images in the book were included, except those that consisted of “conceptual” or abstract works where no anatomically identifiable face was presented. Because some images were printed twice, all were double-checked to insure that all were counted only once, yielding a total of 214 images. These were classified as showing the left side (88), right side (106), or as frontal (20) by the first author. The classification was straightforward as none of the self-portraits was ambiguous in the posing choice, and very few were frontal.

Overall, inspection of Figure 1a confirms a small, but consistent right bias in the side of the face shown on the canvas. A large bias in found in one study that examined only self-portraits by Rembrandt [1], but this is likely to reflect the idiosyncrasies of this specific artist (see also [8]). Further analysis suggested that the bias tends to be strong in the 17th, 18th, and 19th centuries, but weak (and in the opposite direction) before and afterward (see Figure 1b, the huge bias recorded in the 12–14th century window should be interpreted with caution as only seven self- portraits were available in this time window). A temporal dependency of the right-side bias is roughly consistent with an earlier report [2] and strongly suggestive that mirrors may be a part of the explanation for side biases. Mirrors made of polished metal surfaces were available already in the antiquity, and the technique for producing plane mirrors with plated glass surfaces was introduced in Venice around the 14th century. These however were small and expensive, which might have made them less readily available to artists and not suitable for making a full body self-portrait. Techniques for producing larger and cheaper glass plates were gradually introduced only in the 17th century [9]. Assuming that artists have a bias for showing their left- side, this would predict a relative majority of left-sided poses if most drew by memory but a sudden switch to a right-sided preference once most were using mirrors. The invention of photography in mid 19th century [10] obviously implied the brand new possibility of drawing one’s self-portrait from a photograph, although some artists may have continued to use mirrors. These two groups would counterbalance somewhat, cancelling out biases on the average. A recent analysis [11] comparing self-portraits before and after the availability of cameras confirms this interpretation.

However, mirrors could explain the right-side bias in at least two different ways. Artists may be taught to place the mirror on the left of the canvas (as in Figure 1a, inset), a position that should be more natural for a right-handed artist as this avoids occlusion of the reflected image by the arm holding the brush. Such studio conventions and the related biomechanical constraints might well be responsible for a small group bias. As an alternative, the bias may reflect biologically determined asymmetries in facial expressiveness [7]. There is evidence suggesting right-hemispheric dominance for emotional expression [12], which may cause most artists to present their left, more expressive side, to the mirror. When copying the mirror image, these artists would then paint a right-sided face. Distinguishing between these two possibilities would require precise information on artists’ handedness and on the studio arrangements for each analyzed self-portrait, and a large sample. In support of a role for the asymmetry in facial expressiveness, there is evidence for a left- rather than right-side bias when artists compose portraits rather than self-portraits [13]. However, this bias may also arise from biomechanical constraints related to the natural swing of the arm and the direction of the main features of the face as drawn on the canvas [14]. This problem does not apply to a study of photographic portraits in two college yearbooks [15]. Even in this dataset, however, information on how the pictures were taken and later selected for inclusion in the yearbooks would be needed to interpret the results. In addition, a crucial prediction of the facial expressiveness hypothesis is that the bias should be observable even in non-artists. Testing this prediction with paintings is clearly difficult, as non-artists would lack the required technical skills.

We conducted a study of self-portraits by non-artists using the iPhone™ front camera in controlled settings. When using the front camera, the iPhone™ preview display presents a mirror image of the camera view. Thus, our task was representative of what artists do when composing a self-portrait using a mirror, although for our participants this involved a simple button press rather than sophisticated brushwork. The phone then saves the picture in the non-reversed version, that is, as if taken by a photographer facing the participant. Participants explored different poses and recorded their self-portrait once they had found one that they liked. We recorded the participant sex and handedness, and asked whether they had tried making a front- camera self-portrait before. Finally, we showed them the recorded picture and asked to rate it on a 1–7 scale (1 meaning that they did not like it at all, 4 meaning that it was neither good nor bad, and 7 meaning that they liked it very much). This last question was added as an exploratory test of pose preferences in analogy to what would happen if a photographer presented a series of photographs and one had to pick the one that will go into the yearbook. There were three conditions: portrait (phone held vertically, front camera on top, almost centered but slightly offset to the left), landscape left (phone held horizontally, camera on the left of the participant) and landscape right (same as before but camera on the right). This manipulation therefore simulated different mirror positions relative to the artist, allowing us to evaluate the impact of this specific factor.

## Methods

### Participants

A total of 300 participants volunteered. They were recruited within the communities of the Universities of Parma, Bologna, Naples “S.Orsola Benincasa”, and Liverpool. The majority were full-time students (218, plus 6 graduate students) but teaching (25) and administrative staff (10) were also included. An additional 41 individuals were student-workers who were employed in various professional positions. The majority were women (57%) and only 13% were left-handed.

### Design

Participants were assigned at random to one of three conditions (100 participants each). In the portrait condition, they were instructed to hold the phone vertically such that the front camera was on top, and only slightly offset to the left of the display center. In this condition they used the thumb of the right hand to record the picture. In the landscape right condition, they held the phone horizontally with the front camera on their right, and recorded the picture using the thumb of the left hand. In the landscape left condition, they held the phone horizontally with the front camera on their left, and recorded the picture using the thumb of the right hand.

### Procedure

All data used in the study were analyzed anonymously. The research was conducted in accordance with the ethical standards of the Italian Board or Psychologists (see http://www.psy.it/codice_deontologico.html) and of the Italian Psychological Society (AIP, see http://www.aipass.org/node/26). Given that the experiment did not involve clinical tests, use of pharmaceuticals or medical equipment, did not involve the use of deception or involve participant discomfort in any other way, approval of Ethics Committee for Clinical Research of the University of Parma was deemed unnecessary.

Participants read and signed an informed-consent form. This explained the task and asked them to give permission to record their self-portrait for further analysis. Informed consent forms were stored separately from all other information regarding participants to preserve anonymity. Once participants had consented, the experimenter illustrated the task again. We emphasized that there was no right or wrong answer to the task, and that we were only interested in assessing which pose individuals would spontaneously choose when making a self-portrait with a smartphone. However, we explicitly asked all participants to try out different poses, including three quarter poses, before deciding which one they liked best. Only one picture was allowed. When more than one was recorded by mistake we asked participants to indicate which one was the intended one and deleted the others. Once they had recorded the picture, we entered information on their sex, occupation, handedness, and previous experience with front-camera portraits. Finally, we showed them the saved photographs (no longer mirror reversed) and asked them to rate it on a 1–7 scale (1 meaning that they did not like it at all, 4 meaning that it was neither good nor bad, and 7 meaning that they liked it very much). This completed their participation. Care was taken to insure that participants positioned themselves against an approximately homogeneous background, such as a wall, when taking the pictures. We also tried to have approximately homogeneous illumination of both sides of the face, but a post-hoc analysis of the pictures revealed that although this was true in the majority (125) of the pictures, the illumination was in fact slightly stronger on the left side of the participants’ face in 73 pictures and on the right side in 102 pictures. However, in both these subsets the “left” pose remained more frequent than the “right” one (about 30% vs. 23% in both cases). We can therefore rule out that any observed bias was due to a direction of illumination confound.

### Classification of the Recorded Pictures

All data were analyzed anonymously. Self-portraits were parsed into five posing categories: left, right, slight left, slight right, and frontal. The classification was performed in the following way. All pictures were imported into the Apple - iPhoto software and inspected individually on an Apple LCD Cinema Display (30′′ flat panel) monitor. This is a widescreen monitor and the size of the picture within the iPhoto window was approximately 15 cm by 20 cm (portrait) or 20 cm by 15 cm (landscape). If the picture involved a three-quarter pose clearly and unambiguously presenting one side of the face to the camera, we classified the self-portrait as “left” or “right” as was appropriate. If the pose could not be immediately classified by eye, we used a ruler to measure the imaginary horizontal line between the left and right cheek passing through the center of the nose, and determined the lengths of its left and right portions. If there was a difference between these two portions that was larger than the ruler resolution (1 mm), we classified the self-portrait accordingly as “slight left” or “slight right”. All remaining portraits were classified as “frontal”.

## Results and Discussion

**Figure 2:**
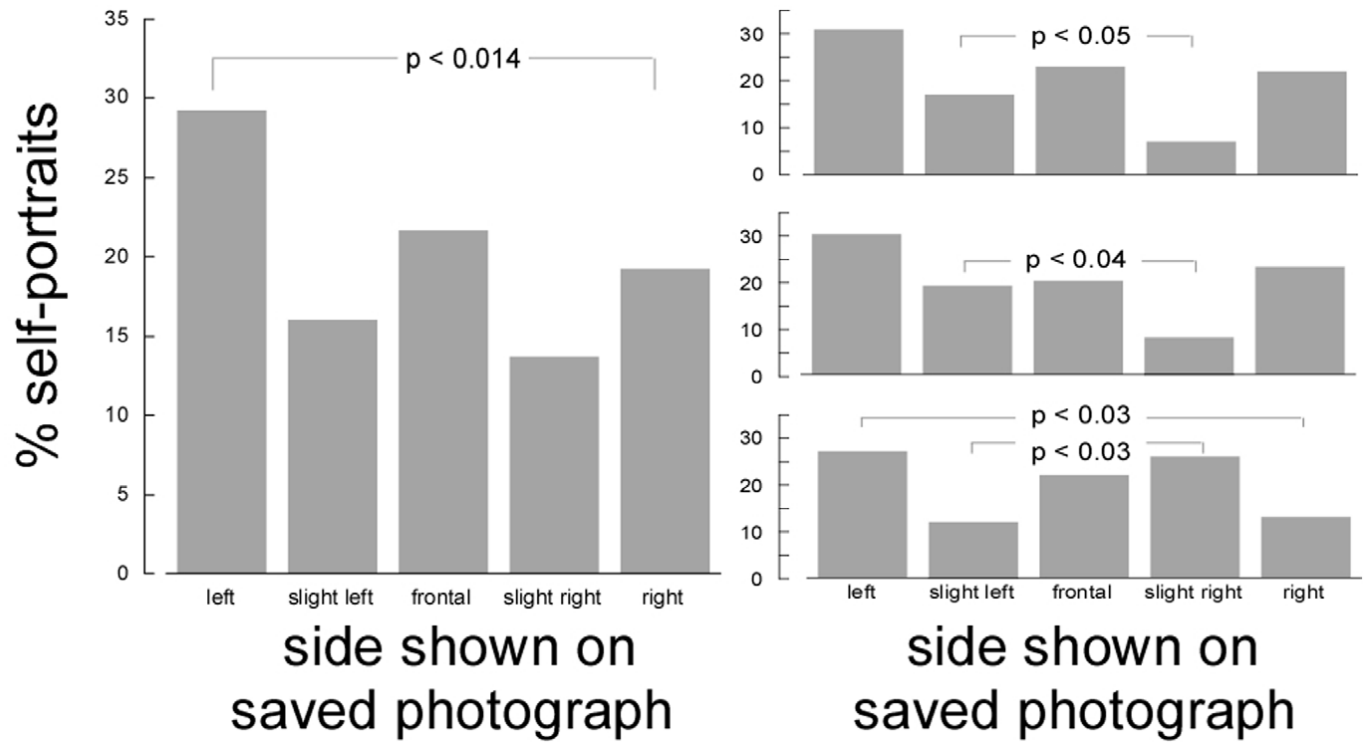
Percentage of five categories of self-portrait, after pooling across three conditions (left) or separately by condition (top: portrait; middle: landscape left camera; bottom: landscape right). Recall that categories refer to the saved photograph (no longer mirror-reversed).

Figure 2a presents the distribution of poses in the saved photographs, pooled across the three conditions. Almost 30% of the self-portraits (88 out of 300) were three-quarter poses showing the left side (recall that this appeared as the right side in the preview display when participants recorded the photograph). Conversely, only about 19% (58) showed the right side. This difference was statistically significant when compared with the frequencies expected for a rectangular distribution, chi- square(1) = 6.16, p<0.014. The side bias was remarkably stable across sexes (23% vs 13% and 34% vs 24% for males and females, respectively), handedness (29% vs 21% and 32% vs 11% for right- and left-handers), and previous experience with the front-camera (31% vs 21% and 28% vs 18% for participants who had and had not tried it before).

The main side bias in the unambiguously rotated portraits remained visible after dividing the self-portraits according to camera orientation (Figure 2b), although not all differences in observed frequencies remained statistically significant due to the reduction in statistical power. However, we continued to observe a significant advantage for the left side in the landscape, camera right group, chi- square(1) = 4.9, p<0.03. Most importantly, when pooling the two landscape groups, we also continued to observe a significant advantage for the left side, chi-square(1) = 4.7, p<0.03. Given that such pooling counterbalances the effect of camera position, if this was the only factor determining side-biases we would expect no bias here but instead we continued to see a left bias. Finally, an effect of camera position emerged when considering the slightly-rotated portraits. In the landscape, camera right group we observed a preference for slight right poses over slight left, chi-square(1) = 5.2, p<0.03. In the landscape, camera left group we instead observed a preference for slight left over slight right poses, chi-square(1) = 4.5, p<0.04. In the portrait group, finally, we also observed a preference for slight left over slight right poses, chi- square(1) = 4.2, p<0.05.

This pattern of results suggests that the position of the camera, our proxy for the mirror position in the artist’s studio, did have a biasing effect on posing choices. When the self-portrait was taken with the “mirror” on the left, this made it more likely that the saved photograph would present the left side of the face (even though this had appeared as a right side in the mirror-reversed display). When the self-portrait was taken with the “mirror” on the right, the opposite bias occurred. Note that camera orientation in our experiment also implied that a different hand was used to take the photo, creating a clear motor asymmetry. However, these motor and viewpoint asymmetries only affected participants that preferred almost-frontal poses. In participants that preferred three-quarter poses (49% of our sample), we continued to observe a preference for left-sided poses in all camera positions, including those that promoted a right-sided bias in slightly rotated poses.

**Figure 3:**
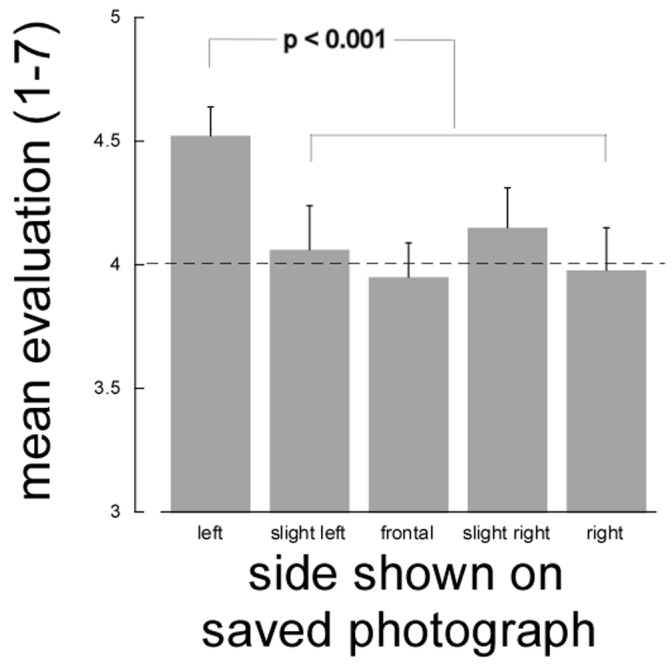
Average participants ratings of their saved photograph (no longer mirror reversed), in each of the five posing categories. Error bars are standard errors of the mean.

Unexpectedly, further confirmation of a difference between the left and right sides emerged from analyzing ratings of the saved photographs. In these, the orientation of the face was the opposite of that in the front-camera preview display. However, such left-right reversals can easily go undetected [16]. In general, images tend to retain their meaning and value when reversed [17] although reversed images may appear subtly different in expressiveness [18]. We expected, therefore, that participants would not be negatively affected by the reversal of the saved picture relative to the image seen when taking the self-portrait. For all categories of portrait the average rating was never less than 4 (the “neither good nor bad” point on the scale). In fact, it was essentially identical to 4 for all categories (3.9<rating<4.1, see Fig. 3), except for the self-portraits unambiguously showing the left side. In this case, the average rating (4.5±0.1) was clearly above 4 and a test contrasting these ratings with all the others was statistically significant, F(1,196) = 11.8, p<0.001. This finding is consistent with the notion that the left side of the face tends to be more expressive. However, because we did not perform a comparison with evaluations of the original views in the preview orientation, this conclusion remains tentative.

Our results demonstrate that a preference for self-portraits showing one’s left side, even if this appears as a right cheek in the mirror-reversed display, can be documented in non- artists. Crucially, this preference can be demonstrated with a controlled procedure that allowed us to evaluate the importance of camera position in determining this bias. Thus our results can be interpreted as evidence for two separate factors affecting asymmetries in facial expressiveness, a posing bias due to the position of the mirror and an actual difference between the two sides of the face. There are other examples, such as the case of a preference for lighting coming from above left, where an asymmetry studied in the laboratory is consistent with one found within the visual arts [19]. Future studies exploiting the flexibility and ease of collecting smartphone photographs may open up new avenues for testing hypotheses in the empirical investigation of the arts. An interesting issue in this respect would be to test photographic portraits using modified devices that do not mirror-reverse the preview display.
